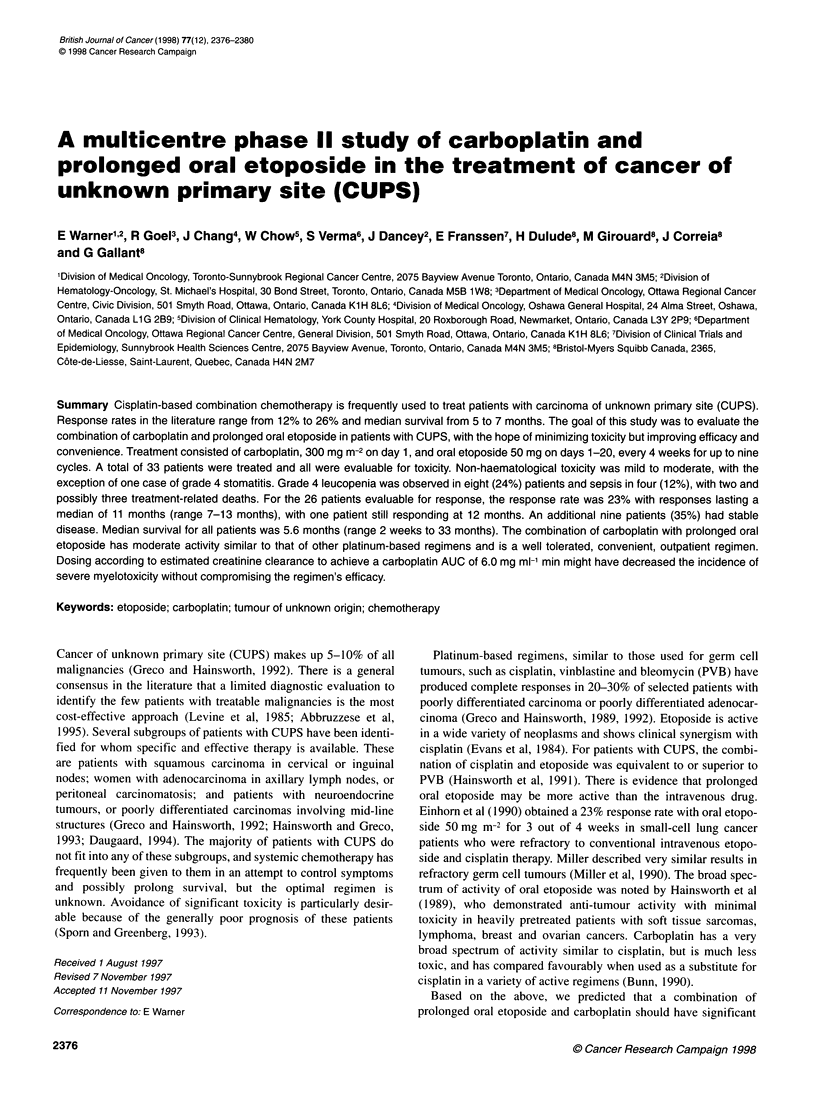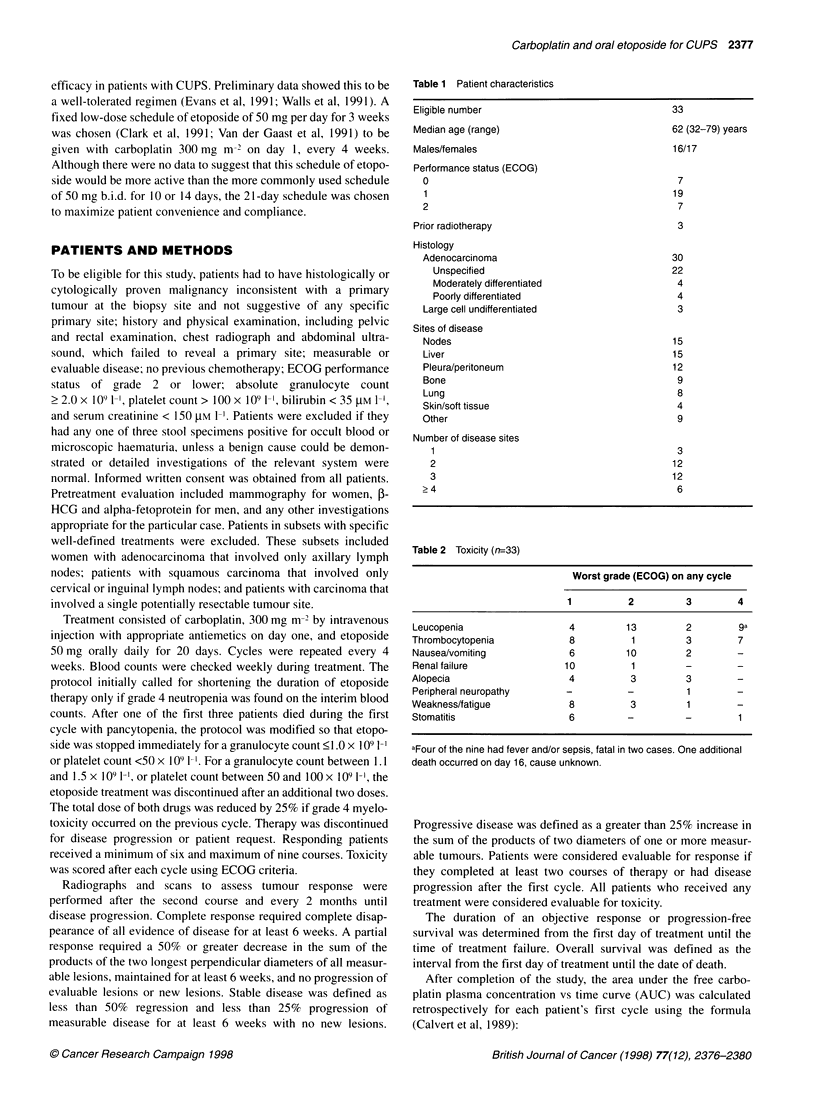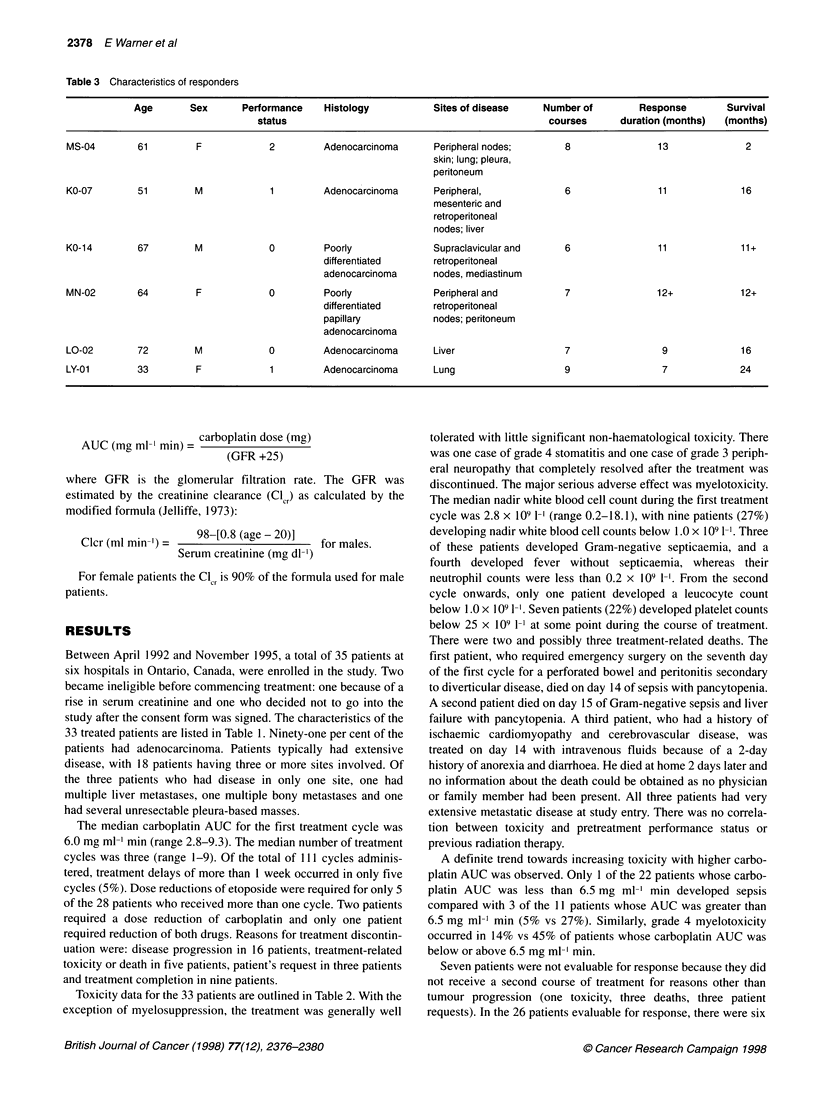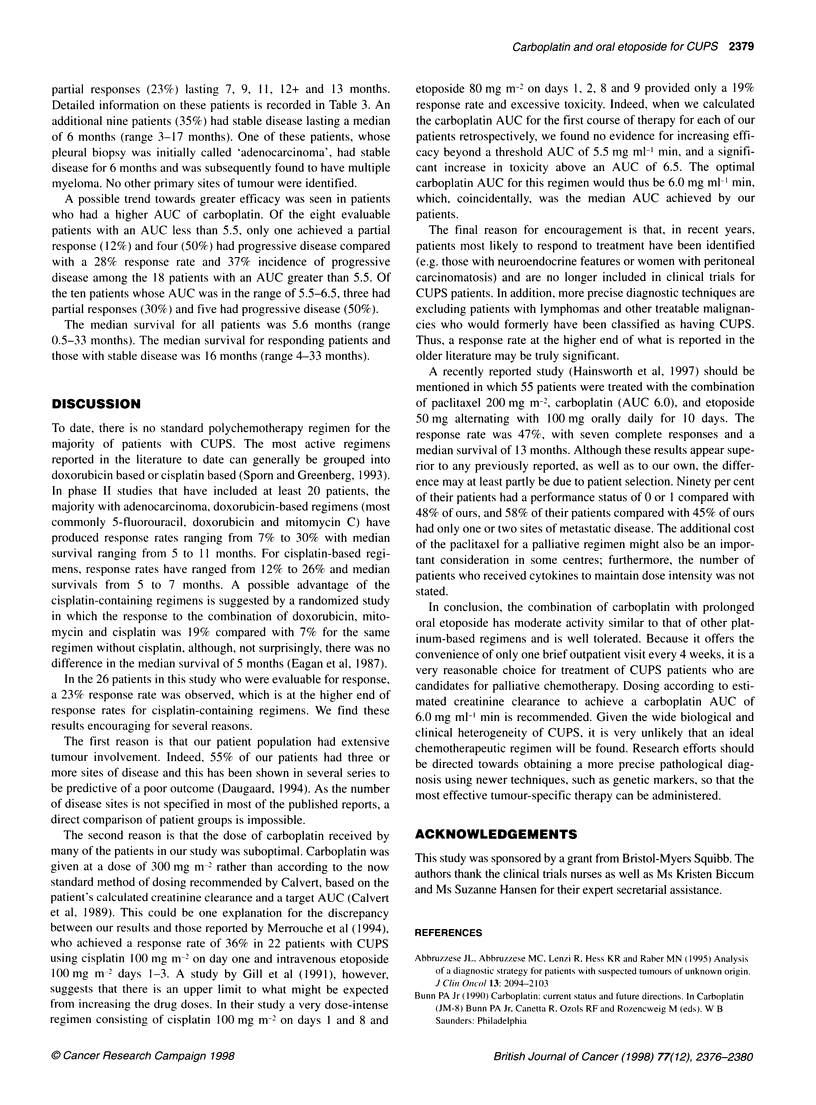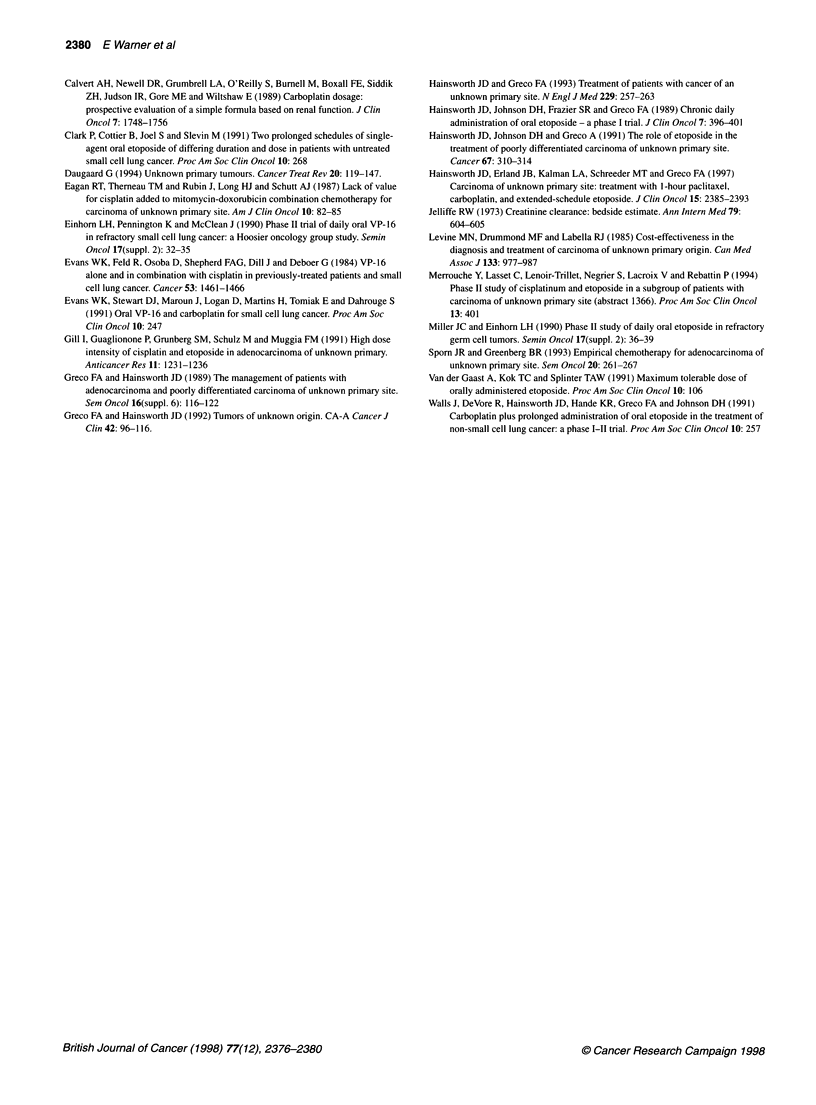# A multicentre phase II study of carboplatin and prolonged oral etoposide in the treatment of cancer of unknown primary site (CUPS).

**DOI:** 10.1038/bjc.1998.395

**Published:** 1998-06

**Authors:** E. Warner, R. Goel, J. Chang, W. Chow, S. Verma, J. Dancey, E. Franssen, H. Dulude, M. Girouard, J. Correia, G. Gallant

**Affiliations:** Division of Medical Oncology, Toronto-Sunnybrook Regional Cancer Centre, Ontario, Canada.

## Abstract

Cisplatin-based combination chemotherapy is frequently used to treat patients with carcinoma of unknown primary site (CUPS). Response rates in the literature range from 12% to 26% and median survival from 5 to 7 months. The goal of this study was to evaluate the combination of carboplatin and prolonged oral etoposide in patients with CUPS, with the hope of minimizing toxicity but improving efficacy and convenience. Treatment consisted of carboplatin, 300 mg m(-2) on day 1, and oral etoposide 50 mg on days 1-20, every 4 weeks for up to nine cycles. A total of 33 patients were treated and all were evaluable for toxicity. Non-haematological toxicity was mild to moderate, with the exception of one case of grade 4 stomatitis. Grade 4 leucopenia was observed in eight (24%) patients and sepsis in four (12%), with two and possibly three treatment-related deaths. For the 26 patients evaluable for response, the response rate was 23% with responses lasting a median of 11 months (range 7-13 months), with one patient still responding at 12 months. An additional nine patients (35%) had stable disease. Median survival for all patients was 5.6 months (range 2 weeks to 33 months). The combination of carboplatin with prolonged oral etoposide has moderate activity similar to that of other platinum-based regimens and is a well tolerated, convenient, outpatient regimen. Dosing according to estimated creatinine clearance to achieve a carboplatin AUC of 6.0 mg ml(-1) min might have decreased the incidence of severe myelotoxicity without compromising the regimen's efficacy.


British Journal of Cancer (1998) 77(12), 2376–2380
© 1998 Cancer Research Campaign

### A multicentre phase II study of carboplatin and prolonged oral etoposide in the treatment of cancer of unknown primary site (CUPS)

**E Warner[1,2], R Goel[3], J Chang[4], W Chow[5], S Verma[6], J Dancey[2], E Franssen[7], H Dulude[8], M Girouard[8], J Correia[8] and G Gallant[8]**

[1]Division of Medical Oncology, Toronto-Sunnybrook Regional Cancer Centre, 2075 Bayview Avenue Toronto, Ontario, Canada M4N 3M5; [2]Division of Hematology-Oncology, St. Michael's Hospital, 30 Bond Street, Toronto, Ontario, Canada M5B 1W8; [3]Department of Medical Oncology, Ottawa Regional Cancer Centre, Civic Division, 501 Smyth Road, Ottawa, Ontario, Canada K1H 8L6; [4]Division of Medical Oncology, Oshawa General Hospital, 24 Alma Street, Oshawa, Ontario, Canada L1G 2B9; [5]Division of Clinical Hematology, York County Hospital, 20 Roxborough Road, Newmarket, Ontario, Canada L3Y 2P9; [6]Department of Medical Oncology, Ottawa Regional Cancer Centre, General Division, 501 Smyth Road, Ottawa, Ontario, Canada K1H 8L6; [7]Division of Clinical Trials and Epidemiology, Sunnybrook Health Sciences Centre, 2075 Bayview Avenue, Toronto, Ontario, Canada M4N 3M5; [8]Bristol-Myers Squibb Canada, 2365, Côte-de-Liesse, Saint-Laurent, Quebec, Canada H4N 2M7

**Summary** Cisplatin-based combination chemotherapy is frequently used to treat patients with carcinoma of unknown primary site (CUPS). Response rates in the literature range from 12% to 26% and median survival from 5 to 7 months. The goal of this study was to evaluate the combination of carboplatin and prolonged oral etoposide in patients with CUPS, with the hope of minimizing toxicity but improving efficacy and convenience. Treatment consisted of carboplatin, 300 mg $m^{-2}$ on day 1, and oral etoposide 50 mg on days 1–20, every 4 weeks for up to nine cycles. A total of 33 patients were treated and all were evaluable for toxicity. Non-haematological toxicity was mild to moderate, with the exception of one case of grade 4 stomatitis. Grade 4 leucopenia was observed in eight (24%) patients and sepsis in four (12%), with two and possibly three treatment-related deaths. For the 26 patients evaluable for response, the response rate was 23% with responses lasting a median of 11 months (range 7–13 months), with one patient still responding at 12 months. An additional nine patients (35%) had stable disease. Median survival for all patients was 5.6 months (range 2 weeks to 33 months). The combination of carboplatin with prolonged oral etoposide has moderate activity similar to that of other platinum-based regimens and is a well tolerated, convenient, outpatient regimen. Dosing according to estimated creatinine clearance to achieve a carboplatin AUC of 6.0 mg $ml^{-1}$ min might have decreased the incidence of severe myelotoxicity without compromising the regimen's efficacy.

**Keywords:** etoposide; carboplatin; tumour of unknown origin; chemotherapy

Cancer of unknown primary site (CUPS) makes up 5–10% of all malignancies (Greco and Hainsworth, 1992). There is a general consensus in the literature that a limited diagnostic evaluation to identify the few patients with treatable malignancies is the most cost-effective approach (Levine et al, 1985; Abbruzzese et al, 1995). Several subgroups of patients with CUPS have been identified for whom specific and effective therapy is available. These are patients with squamous carcinoma in cervical or inguinal nodes; women with adenocarcinoma in axillary lymph nodes, or peritoneal carcinomatosis; and patients with neuroendocrine tumours, or poorly differentiated carcinomas involving mid-line structures (Greco and Hainsworth, 1992; Hainsworth and Greco, 1993; Daugaard, 1994). The majority of patients with CUPS do not fit into any of these subgroups, and systemic chemotherapy has frequently been given to them in an attempt to control symptoms and possibly prolong survival, but the optimal regimen is unknown. Avoidance of significant toxicity is particularly desirable because of the generally poor prognosis of these patients (Sporn and Greenberg, 1993).

*Received 1 August 1997*
*Revised 7 November 1997*
*Accepted 11 November 1997*

*Correspondence to:* E Warner

Platinum-based regimens, similar to those used for germ cell tumours, such as cisplatin, vinblastine and bleomycin (PVB) have produced complete responses in 20–30% of selected patients with poorly differentiated carcinoma or poorly differentiated adenocarcinoma (Greco and Hainsworth, 1989, 1992). Etoposide is active in a wide variety of neoplasms and shows clinical synergism with cisplatin (Evans et al, 1984). For patients with CUPS, the combination of cisplatin and etoposide was equivalent to or superior to PVB (Hainsworth et al, 1991). There is evidence that prolonged oral etoposide may be more active than the intravenous drug. Einhorn et al (1990) obtained a 23% response rate with oral etoposide 50 mg $m^{-2}$ for 3 out of 4 weeks in small-cell lung cancer patients who were refractory to conventional intravenous etoposide and cisplatin therapy. Miller described very similar results in refractory germ cell tumours (Miller et al, 1990). The broad spectrum of activity of oral etoposide was noted by Hainsworth et al (1989), who demonstrated anti-tumour activity with minimal toxicity in heavily pretreated patients with soft tissue sarcomas, lymphoma, breast and ovarian cancers. Carboplatin has a very broad spectrum of activity similar to cisplatin, but is much less toxic, and has compared favourably when used as a substitute for cisplatin in a variety of active regimens (Bunn, 1990).

Based on the above, we predicted that a combination of prolonged oral etoposide and carboplatin should have significant

© Cancer Research Campaign 1998

efficacy in patients with CUPS. Preliminary data showed this to be a well-tolerated regimen (Evans et al, 1991; Walls et al, 1991). A fixed low-dose schedule of etoposide of 50 mg per day for 3 weeks was chosen (Clark et al, 1991; Van der Gaast et al, 1991) to be given with carboplatin 300 mg $m^{-2}$ on day 1, every 4 weeks. Although there were no data to suggest that this schedule of etoposide would be more active than the more commonly used schedule of 50 mg b.i.d. for 10 or 14 days, the 21-day schedule was chosen to maximize patient convenience and compliance.

### PATIENTS AND METHODS

To be eligible for this study, patients had to have histologically or cytologically proven malignancy inconsistent with a primary tumour at the biopsy site and not suggestive of any specific primary site; history and physical examination, including pelvic and rectal examination, chest radiograph and abdominal ultrasound, which failed to reveal a primary site; measurable or evaluable disease; no previous chemotherapy; ECOG performance status of grade 2 or lower; absolute granulocyte count $\geq 2.0 \times 10^9$ $l^{-1}$, platelet count > $100 \times 10^9$ $l^{-1}$, bilirubin < 35 μM $l^{-1}$, and serum creatinine < 150 μM $l^{-1}$. Patients were excluded if they had any one of three stool specimens positive for occult blood or microscopic haematuria, unless a benign cause could be demonstrated or detailed investigations of the relevant system were normal. Informed written consent was obtained from all patients. Pretreatment evaluation included mammography for women, β-HCG and alpha-fetoprotein for men, and any other investigations appropriate for the particular case. Patients in subsets with specific well-defined treatments were excluded. These subsets included women with adenocarcinoma that involved only axillary lymph nodes; patients with squamous carcinoma that involved only cervical or inguinal lymph nodes; and patients with carcinoma that involved a single potentially resectable tumour site.

Treatment consisted of carboplatin, 300 mg $m^{-2}$ by intravenous injection with appropriate antiemetics on day one, and etoposide 50 mg orally daily for 20 days. Cycles were repeated every 4 weeks. Blood counts were checked weekly during treatment. The protocol initially called for shortening the duration of etoposide therapy only if grade 4 neutropenia was found on the interim blood counts. After one of the first three patients died during the first cycle with pancytopenia, the protocol was modified so that etoposide was stopped immediately for a granulocyte count $\leq 1.0 \times 10^9$ $l^{-1}$ or platelet count $< 50 \times 10^9$ $l^{-1}$. For a granulocyte count between 1.1 and $1.5 \times 10^9$ $l^{-1}$, or platelet count between 50 and $100 \times 10^9$ $l^{-1}$, the etoposide treatment was discontinued after an additional two doses. The total dose of both drugs was reduced by 25% if grade 4 myelotoxicity occurred on the previous cycle. Therapy was discontinued for disease progression or patient request. Responding patients received a minimum of six and maximum of nine courses. Toxicity was scored after each cycle using ECOG criteria.

Radiographs and scans to assess tumour response were performed after the second course and every 2 months until disease progression. Complete response required complete disappearance of all evidence of disease for at least 6 weeks. A partial response required a 50% or greater decrease in the sum of the products of the two longest perpendicular diameters of all measurable lesions, maintained for at least 6 weeks, and no progression of evaluable lesions or new lesions. Stable disease was defined as less than 50% regression and less than 25% progression of measurable disease for at least 6 weeks with no new lesions.

**Table 1** Patient characteristics

| | |
|---|---|
| Eligible number | 33 |
| Median age (range) | 62 (32–79) years |
| Males/females | 16/17 |
| Performance status (ECOG) | |
| 0 | 7 |
| 1 | 19 |
| 2 | 7 |
| Prior radiotherapy | 3 |
| Histology | |
| Adenocarcinoma | 30 |
| Unspecified | 22 |
| Moderately differentiated | 4 |
| Poorly differentiated | 4 |
| Large cell undifferentiated | 3 |
| Sites of disease | |
| Nodes | 15 |
| Liver | 15 |
| Pleura/peritoneum | 12 |
| Bone | 9 |
| Lung | 8 |
| Skin/soft tissue | 4 |
| Other | 9 |
| Number of disease sites | |
| 1 | 3 |
| 2 | 12 |
| 3 | 12 |
| ≥ 4 | 6 |

**Table 2** Toxicity (*n*=33)

| | Worst grade (ECOG) on any cycle | | | |
|---|---|---|---|---|
| | 1 | 2 | 3 | 4 |
| Leucopenia | 4 | 13 | 2 | 9[a] |
| Thrombocytopenia | 8 | 1 | 3 | 7 |
| Nausea/vomiting | 6 | 10 | 2 | – |
| Renal failure | 10 | 1 | – | – |
| Alopecia | 4 | 3 | 3 | – |
| Peripheral neuropathy | – | – | 1 | – |
| Weakness/fatigue | 8 | 3 | 1 | – |
| Stomatitis | 6 | – | – | 1 |

[a]Four of the nine had fever and/or sepsis, fatal in two cases. One additional death occurred on day 16, cause unknown.

Progressive disease was defined as a greater than 25% increase in the sum of the products of two diameters of one or more measurable tumours. Patients were considered evaluable for response if they completed at least two courses of therapy or had disease progression after the first cycle. All patients who received any treatment were considered evaluable for toxicity.

The duration of an objective response or progression-free survival was determined from the first day of treatment until the time of treatment failure. Overall survival was defined as the interval from the first day of treatment until the date of death.

After completion of the study, the area under the free carboplatin plasma concentration vs time curve (AUC) was calculated retrospectively for each patient's first cycle using the formula (Calvert et al, 1989):

© Cancer Research Campaign 1998

**Table 3** Characteristics of responders

| | Age | Sex | Performance status | Histology | Sites of disease | Number of courses | Response duration (months) | Survival (months) |
|---|---|---|---|---|---|---|---|---|
| MS-04 | 61 | F | 2 | Adenocarcinoma | Peripheral nodes; skin; lung; pleura, peritoneum | 8 | 13 | 2 |
| K0-07 | 51 | M | 1 | Adenocarcinoma | Peripheral, mesenteric and retroperitoneal nodes; liver | 6 | 11 | 16 |
| K0-14 | 67 | M | 0 | Poorly differentiated adenocarcinoma | Supraclavicular and retroperitoneal nodes, mediastinum | 6 | 11 | 11+ |
| MN-02 | 64 | F | 0 | Poorly differentiated papillary adenocarcinoma | Peripheral and retroperitoneal nodes; peritoneum | 7 | 12+ | 12+ |
| LO-02 | 72 | M | 0 | Adenocarcinoma | Liver | 7 | 9 | 16 |
| LY-01 | 33 | F | 1 | Adenocarcinoma | Lung | 9 | 7 | 24 |

$$\text{AUC (mg ml}^{-1}\text{ min)} = \frac{\text{carboplatin dose (mg)}}{\text{(GFR +25)}}$$

where GFR is the glomerular filtration rate. The GFR was estimated by the creatinine clearance ($Cl_{cr}$) as calculated by the modified formula (Jelliffe, 1973):

$$\text{Clcr (ml min}^{-1}\text{)} = \frac{98-[0.8\text{ (age} - 20)]}{\text{Serum creatinine (mg dl}^{-1}\text{)}} \quad \text{for males.}$$

For female patients the $Cl_{cr}$ is 90% of the formula used for male patients.

### RESULTS

Between April 1992 and November 1995, a total of 35 patients at six hospitals in Ontario, Canada, were enrolled in the study. Two became ineligible before commencing treatment: one because of a rise in serum creatinine and one who decided not to go into the study after the consent form was signed. The characteristics of the 33 treated patients are listed in Table 1. Ninety-one per cent of the patients had adenocarcinoma. Patients typically had extensive disease, with 18 patients having three or more sites involved. Of the three patients who had disease in only one site, one had multiple liver metastases, one multiple bony metastases and one had several unresectable pleura-based masses.

The median carboplatin AUC for the first treatment cycle was 6.0 mg ml$^{-1}$ min (range 2.8–9.3). The median number of treatment cycles was three (range 1–9). Of the total of 111 cycles administered, treatment delays of more than 1 week occurred in only five cycles (5%). Dose reductions of etoposide were required for only 5 of the 28 patients who received more than one cycle. Two patients required a dose reduction of carboplatin and only one patient required reduction of both drugs. Reasons for treatment discontinuation were: disease progression in 16 patients, treatment-related toxicity or death in five patients, patient's request in three patients and treatment completion in nine patients.

Toxicity data for the 33 patients are outlined in Table 2. With the exception of myelosuppression, the treatment was generally well tolerated with little significant non-haematological toxicity. There was one case of grade 4 stomatitis and one case of grade 3 peripheral neuropathy that completely resolved after the treatment was discontinued. The major serious adverse effect was myelotoxicity. The median nadir white blood cell count during the first treatment cycle was $2.8 \times 10^9$ l$^{-1}$ (range 0.2–18.1), with nine patients (27%) developing nadir white blood cell counts below $1.0 \times 10^9$ l$^{-1}$. Three of these patients developed Gram-negative septicaemia, and a fourth developed fever without septicaemia, whereas their neutrophil counts were less than $0.2 \times 10^9$ l$^{-1}$. From the second cycle onwards, only one patient developed a leucocyte count below $1.0 \times 10^9$ l$^{-1}$. Seven patients (22%) developed platelet counts below $25 \times 10^9$ l$^{-1}$ at some point during the course of treatment. There were two and possibly three treatment-related deaths. The first patient, who required emergency surgery on the seventh day of the first cycle for a perforated bowel and peritonitis secondary to diverticular disease, died on day 14 of sepsis with pancytopenia. A second patient died on day 15 of Gram-negative sepsis and liver failure with pancytopenia. A third patient, who had a history of ischaemic cardiomyopathy and cerebrovascular disease, was treated on day 14 with intravenous fluids because of a 2-day history of anorexia and diarrhoea. He died at home 2 days later and no information about the death could be obtained as no physician or family member had been present. All three patients had very extensive metastatic disease at study entry. There was no correlation between toxicity and pretreatment performance status or previous radiation therapy.

A definite trend towards increasing toxicity with higher carboplatin AUC was observed. Only 1 of the 22 patients whose carboplatin AUC was less than 6.5 mg ml$^{-1}$ min developed sepsis compared with 3 of the 11 patients whose AUC was greater than 6.5 mg ml$^{-1}$ min (5% vs 27%). Similarly, grade 4 myelotoxicity occurred in 14% vs 45% of patients whose carboplatin AUC was below or above 6.5 mg ml$^{-1}$ min.

Seven patients were not evaluable for response because they did not receive a second course of treatment for reasons other than tumour progression (one toxicity, three deaths, three patient requests). In the 26 patients evaluable for response, there were six

 © Cancer Research Campaign 1998

partial responses (23%) lasting 7, 9, 11, 12+ and 13 months. Detailed information on these patients is recorded in Table 3. An additional nine patients (35%) had stable disease lasting a median of 6 months (range 3–17 months). One of these patients, whose pleural biopsy was initially called 'adenocarcinoma', had stable disease for 6 months and was subsequently found to have multiple myeloma. No other primary sites of tumour were identified.

A possible trend towards greater efficacy was seen in patients who had a higher AUC of carboplatin. Of the eight evaluable patients with an AUC less than 5.5, only one achieved a partial response (12%) and four (50%) had progressive disease compared with a 28% response rate and 37% incidence of progressive disease among the 18 patients with an AUC greater than 5.5. Of the ten patients whose AUC was in the range of 5.5–6.5, three had partial responses (30%) and five had progressive disease (50%).

The median survival for all patients was 5.6 months (range 0.5–33 months). The median survival for responding patients and those with stable disease was 16 months (range 4–33 months).

### DISCUSSION

To date, there is no standard polychemotherapy regimen for the majority of patients with CUPS. The most active regimens reported in the literature to date can generally be grouped into doxorubicin based or cisplatin based (Sporn and Greenberg, 1993). In phase II studies that have included at least 20 patients, the majority with adenocarcinoma, doxorubicin-based regimens (most commonly 5-fluorouracil, doxorubicin and mitomycin C) have produced response rates ranging from 7% to 30% with median survival ranging from 5 to 11 months. For cisplatin-based regimens, response rates have ranged from 12% to 26% and median survivals from 5 to 7 months. A possible advantage of the cisplatin-containing regimens is suggested by a randomized study in which the response to the combination of doxorubicin, mitomycin and cisplatin was 19% compared with 7% for the same regimen without cisplatin, although, not surprisingly, there was no difference in the median survival of 5 months (Eagan et al, 1987).

In the 26 patients in this study who were evaluable for response, a 23% response rate was observed, which is at the higher end of response rates for cisplatin-containing regimens. We find these results encouraging for several reasons.

The first reason is that our patient population had extensive tumour involvement. Indeed, 55% of our patients had three or more sites of disease and this has been shown in several series to be predictive of a poor outcome (Daugaard, 1994). As the number of disease sites is not specified in most of the published reports, a direct comparison of patient groups is impossible.

The second reason is that the dose of carboplatin received by many of the patients in our study was suboptimal. Carboplatin was given at a dose of 300 mg $m^{-2}$ rather than according to the now standard method of dosing recommended by Calvert, based on the patient's calculated creatinine clearance and a target AUC (Calvert et al, 1989). This could be one explanation for the discrepancy between our results and those reported by Merrouche et al (1994), who achieved a response rate of 36% in 22 patients with CUPS using cisplatin 100 mg $m^{-2}$ on day one and intravenous etoposide 100 mg $m^{-2}$ days 1–3. A study by Gill et al (1991), however, suggests that there is an upper limit to what might be expected from increasing the drug doses. In their study a very dose-intense regimen consisting of cisplatin 100 mg $m^{-2}$ on days 1 and 8 and etoposide 80 mg $m^{-2}$ on days 1, 2, 8 and 9 provided only a 19% response rate and excessive toxicity. Indeed, when we calculated the carboplatin AUC for the first course of therapy for each of our patients retrospectively, we found no evidence for increasing efficacy beyond a threshold AUC of 5.5 mg $ml^{-1}$ min, and a significant increase in toxicity above an AUC of 6.5. The optimal carboplatin AUC for this regimen would thus be 6.0 mg $ml^{-1}$ min, which, coincidentally, was the median AUC achieved by our patients.

The final reason for encouragement is that, in recent years, patients most likely to respond to treatment have been identified (e.g. those with neuroendocrine features or women with peritoneal carcinomatosis) and are no longer included in clinical trials for CUPS patients. In addition, more precise diagnostic techniques are excluding patients with lymphomas and other treatable malignancies who would formerly have been classified as having CUPS. Thus, a response rate at the higher end of what is reported in the older literature may be truly significant.

A recently reported study (Hainsworth et al, 1997) should be mentioned in which 55 patients were treated with the combination of paclitaxel 200 mg $m^{-2}$, carboplatin (AUC 6.0), and etoposide 50 mg alternating with 100 mg orally daily for 10 days. The response rate was 47%, with seven complete responses and a median survival of 13 months. Although these results appear superior to any previously reported, as well as to our own, the difference may at least partly be due to patient selection. Ninety per cent of their patients had a performance status of 0 or 1 compared with 48% of ours, and 58% of their patients compared with 45% of ours had only one or two sites of metastatic disease. The additional cost of the paclitaxel for a palliative regimen might also be an important consideration in some centres; furthermore, the number of patients who received cytokines to maintain dose intensity was not stated.

In conclusion, the combination of carboplatin with prolonged oral etoposide has moderate activity similar to that of other platinum-based regimens and is well tolerated. Because it offers the convenience of only one brief outpatient visit every 4 weeks, it is a very reasonable choice for treatment of CUPS patients who are candidates for palliative chemotherapy. Dosing according to estimated creatinine clearance to achieve a carboplatin AUC of 6.0 mg $ml^{-1}$ min is recommended. Given the wide biological and clinical heterogeneity of CUPS, it is very unlikely that an ideal chemotherapeutic regimen will be found. Research efforts should be directed towards obtaining a more precise pathological diagnosis using newer techniques, such as genetic markers, so that the most effective tumour-specific therapy can be administered.

### ACKNOWLEDGEMENTS

This study was sponsored by a grant from Bristol-Myers Squibb. The authors thank the clinical trials nurses as well as Ms Kristen Biccum and Ms Suzanne Hansen for their expert secretarial assistance.

### REFERENCES

Abbruzzese JL, Abbruzzese MC, Lenzi R, Hess KR and Raber MN (1995) Analysis of a diagnostic strategy for patients with suspected tumours of unknown origin. *J Clin Oncol* **13**: 2094–2103

Bunn PA Jr (1990) Carboplatin: current status and future directions. In Carboplatin (JM-8) Bunn PA Jr, Canetta R, Ozols RF and Rozencweig M (eds). W B Saunders: Philadelphia

© Cancer Research Campaign 1998

Calvert AH, Newell DR, Grumbrell LA, O'Reilly S, Burnell M, Boxall FE, Siddik ZH, Judson IR, Gore ME and Wiltshaw E (1989) Carboplatin dosage: prospective evaluation of a simple formula based on renal function. *J Clin Oncol* **7**: 1748–1756

Clark P, Cottier B, Joel S and Slevin M (1991) Two prolonged schedules of single-agent oral etoposide of differing duration and dose in patients with untreated small cell lung cancer. *Proc Am Soc Clin Oncol* **10**: 268

Daugaard G (1994) Unknown primary tumours. *Cancer Treat Rev* **20**: 119–147.

Eagan RT, Therneau TM and Rubin J, Long HJ and Schutt AJ (1987) Lack of value for cisplatin added to mitomycin-doxorubicin combination chemotherapy for carcinoma of unknown primary site. *Am J Clin Oncol* **10**: 82–85

Einhorn LH, Pennington K and McClean J (1990) Phase II trial of daily oral VP-16 in refractory small cell lung cancer: a Hoosier oncology group study. *Semin Oncol* **17**(suppl. 2): 32–35

Evans WK, Feld R, Osoba D, Shepherd FAG, Dill J and Deboer G (1984) VP-16 alone and in combination with cisplatin in previously-treated patients and small cell lung cancer. *Cancer* **53**: 1461–1466

Evans WK, Stewart DJ, Maroun J, Logan D, Martins H, Tomiak E and Dahrouge S (1991) Oral VP-16 and carboplatin for small cell lung cancer. *Proc Am Soc Clin Oncol* **10**: 247

Gill I, Guaglionone P, Grunberg SM, Schulz M and Muggia FM (1991) High dose intensity of cisplatin and etoposide in adenocarcinoma of unknown primary. *Anticancer Res* **11**: 1231–1236

Greco FA and Hainsworth JD (1989) The management of patients with adenocarcinoma and poorly differentiated carcinoma of unknown primary site. *Sem Oncol* **16**(suppl. 6): 116–122

Greco FA and Hainsworth JD (1992) Tumors of unknown origin. CA-A *Cancer J Clin* **42**: 96–116.

Hainsworth JD and Greco FA (1993) Treatment of patients with cancer of an unknown primary site. *N Engl J Med* **229**: 257–263

Hainsworth JD, Johnson DH, Frazier SR and Greco FA (1989) Chronic daily administration of oral etoposide – a phase I trial. *J Clin Oncol* **7**: 396–401

Hainsworth JD, Johnson DH and Greco A (1991) The role of etoposide in the treatment of poorly differentiated carcinoma of unknown primary site. *Cancer* **67**: 310–314

Hainsworth JD, Erland JB, Kalman LA, Schreeder MT and Greco FA (1997) Carcinoma of unknown primary site: treatment with 1-hour paclitaxel, carboplatin, and extended-schedule etoposide. *J Clin Oncol* **15**: 2385–2393

Jelliffe RW (1973) Creatinine clearance: bedside estimate. *Ann Intern Med* **79**: 604–605

Levine MN, Drummond MF and Labella RJ (1985) Cost-effectiveness in the diagnosis and treatment of carcinoma of unknown primary origin. *Can Med Assoc J* **133**: 977–987

Merrouche Y, Lasset C, Lenoir-Trillet, Negrier S, Lacroix V and Rebattin P (1994) Phase II study of cisplatinum and etoposide in a subgroup of patients with carcinoma of unknown primary site (abstract 1366). *Proc Am Soc Clin Oncol* **13**: 401

Miller JC and Einhorn LH (1990) Phase II study of daily oral etoposide in refractory germ cell tumors. *Semin Oncol* **17**(suppl. 2): 36–39

Sporn JR and Greenberg BR (1993) Empirical chemotherapy for adenocarcinoma of unknown primary site. *Sem Oncol* **20**: 261–267

Van der Gaast A, Kok TC and Splinter TAW (1991) Maximum tolerable dose of orally administered etoposide. *Proc Am Soc Clin Oncol* **10**: 106

Walls J, DeVore R, Hainsworth JD, Hande KR, Greco FA and Johnson DH (1991) Carboplatin plus prolonged administration of oral etoposide in the treatment of non-small cell lung cancer: a phase I–II trial. *Proc Am Soc Clin Oncol* **10**: 257

© Cancer Research Campaign 1998